# Publisher Correction: ComPath: an ecosystem for exploring, analyzing, and curating mappings across pathway databases

**DOI:** 10.1038/s41540-019-0082-7

**Published:** 2019-03-07

**Authors:** Daniel Domingo-Fernández, Charles Tapley Hoyt, Carlos Bobis-Álvarez, Josep Marín-Llaó, Martin Hofmann-Apitius

**Affiliations:** 1 Department of Bioinformatics, Fraunhofer Institute for Algorithms and Scientific Computing, 53754 Sankt Augustin, Germany; 2 Bonn-Aachen International Center for IT, Rheinische Friedrich-Wilhelms-Universität Bonn, 53115 Bonn, Germany; 3 Faculty of Medicine and Health Sciences, University of Oviedo, 33006 Oviedo, Spain; 4 Rovira i Virgili University, 43003 Tarragona, Spain

**Correction to:** *npj Systems Biology and Applications*; 10.1038/s41540-018-0078-8, Published online 13 December 2018

The original version of this Article had an incorrect Article number of 3, an incorrect Volume of 5 and an incorrect Publication year of 2019. These errors have now been corrected in the PDF and HTML versions of the Article.

