# Supplementary material for: ComPath: an ecosystem for exploring, analyzing, and curating mappings across pathway databases
Source: NPJ Syst Biol Appl. 2018 Dec 13;4:43. doi: 10.1038/s41540-018-0078-8 (PMC6292919; doi:10.1038/s41540-018-0078-8)
Supplement: Supplementary file 1 — Supplementary Text [file 41540_2018_78_MOESM1_ESM.pdf]

Supplement

Outline

All the links in this supplement point to resources located at https://github.com/ComPath/resources.

The supplementary information is divided into three sections based on the content: Figures, Tables, and Supplementary Text.

- Figures:
  - Genetic-centric Coverage of Pathway Databases
  - Case Study II: Dendrogram View
  - Visualizing Hierarchical Mappings
  - Pathways Without Mappings in each Database
  - Similarity Landscape of the Curated Mappings
  - Evaluation of Pathway Similarity in the Mapping exercise
- Tables:
  - Resources Loaded in the Deployed ComPath Web Application
  - Statistics Summary of Pathway Sizes across KEGG, Reactome, and WikiPathways
  - Equivalent Pathways within the same Database
  - Equivalent Pathways across KEGG, Reactome, and WikiPathways
  - Curation examples
- Supplementary Text:
  - Case Study II: Additional Findings
  - Software Installation

Figures

Genetic-centric Coverage of Pathway Databases

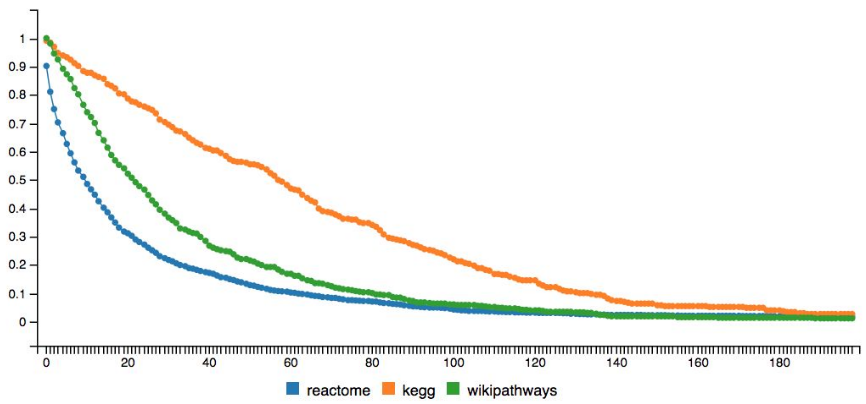

Figure 1. Relative number of pathways in KEGG, Reactome, and WikiPathways with at least X common genes must be present in a pathway. This figure can be interactively visualized at https://compath.scai.fraunhofer.de/simulation and it is described step by stept in this following Jupyter notebook: https://github.com/ComPath/resources/blob/master/notebooks/Gene%20centric%20coverage%20of%20Pathway%20Databases.ipynb

Case Study II: Dendrogram View

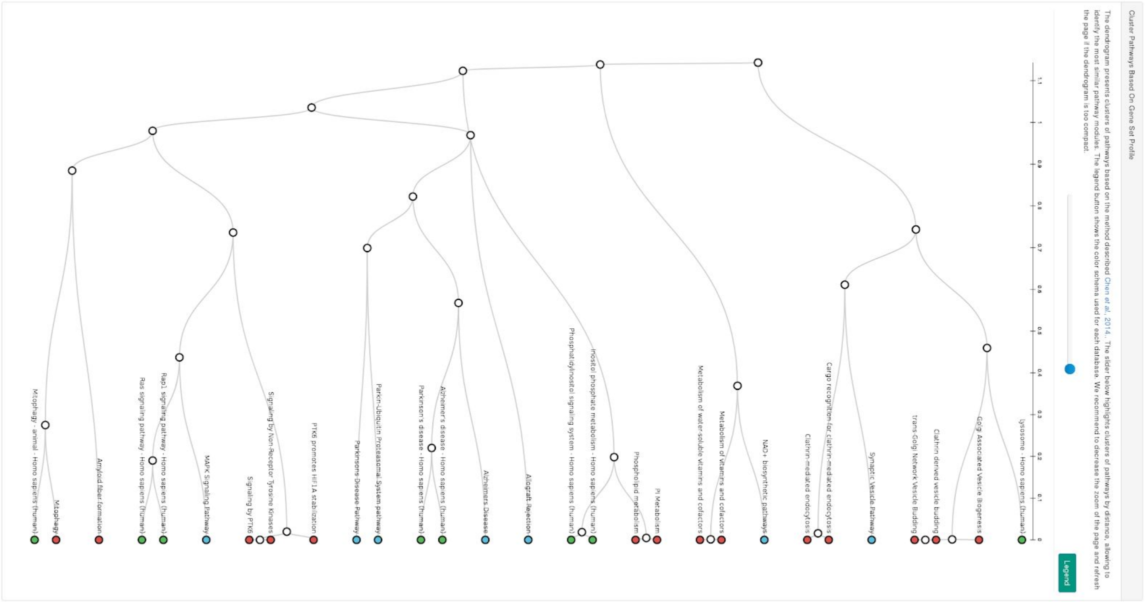

Figure 2. Dendrogram representation of the enriched PDgset pathways hierarchically clustered by their similarity. Link to the interactive visualization here.

Visualizing Hierarchical Mappings

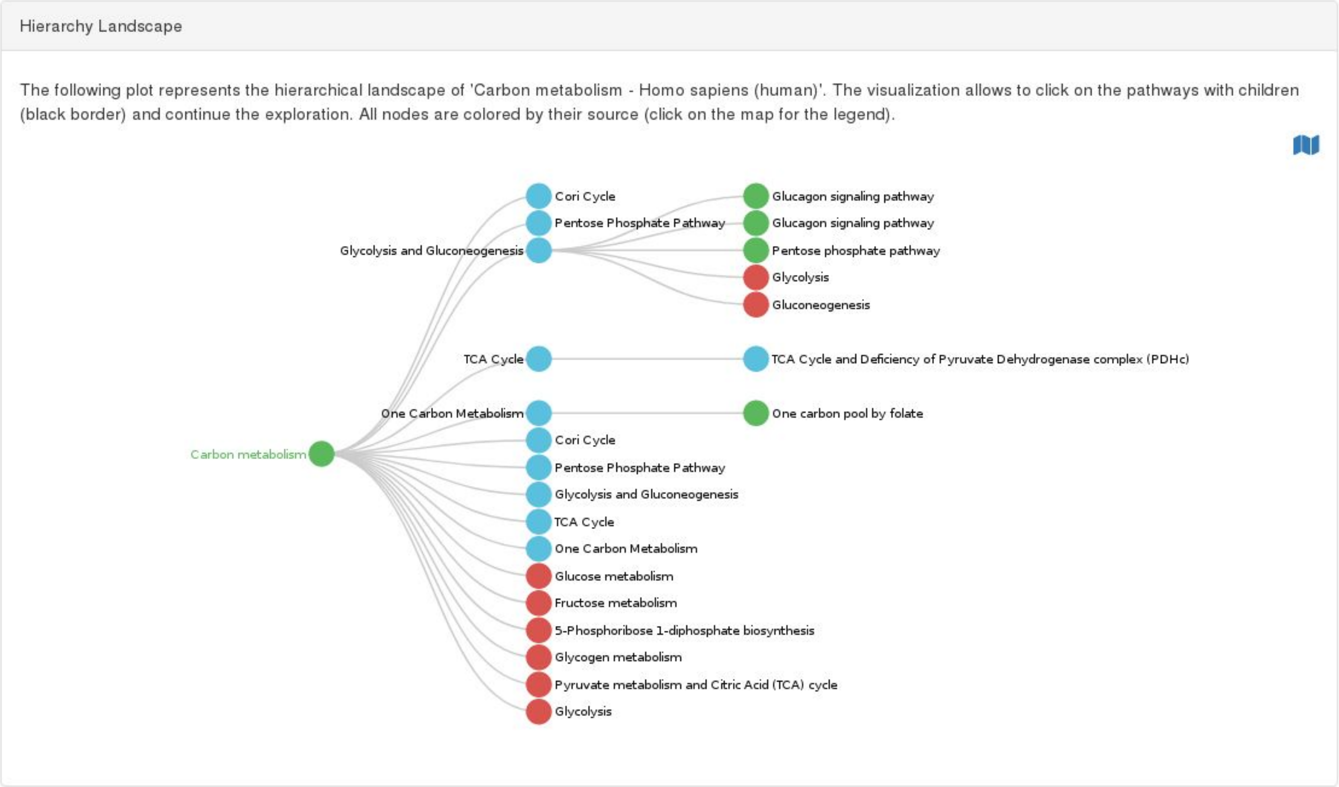

Figure 3. Exploration of the hierarchical mappings of a given pathway. The example rendered the hierarchy of the KEGG pathway *Carbon metabolism*.

Pathways Without Mappings in each Database

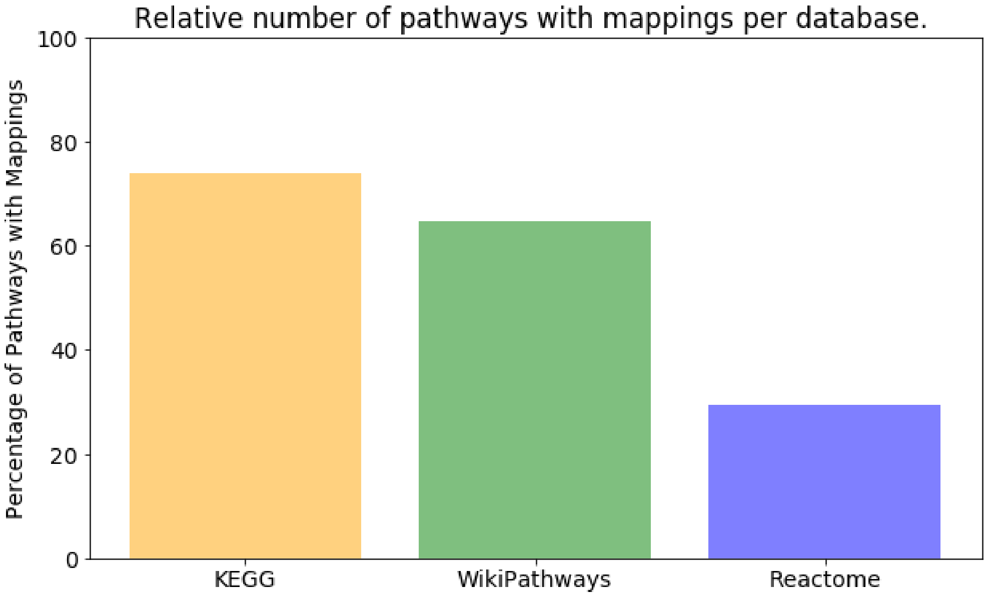

Figure 4. Number of pathways with at least one mapping with respect to the total number of pathways in each database: KEGG, Wikipathways, and Reactome. The Jupyter notebook that outlines this analysis is located at https://github.com/ComPath/resources/blob/master/notebooks/Pathways%20without%20mappings.ipynb

Evaluation of Pathway Similarity in the Mapping exercise

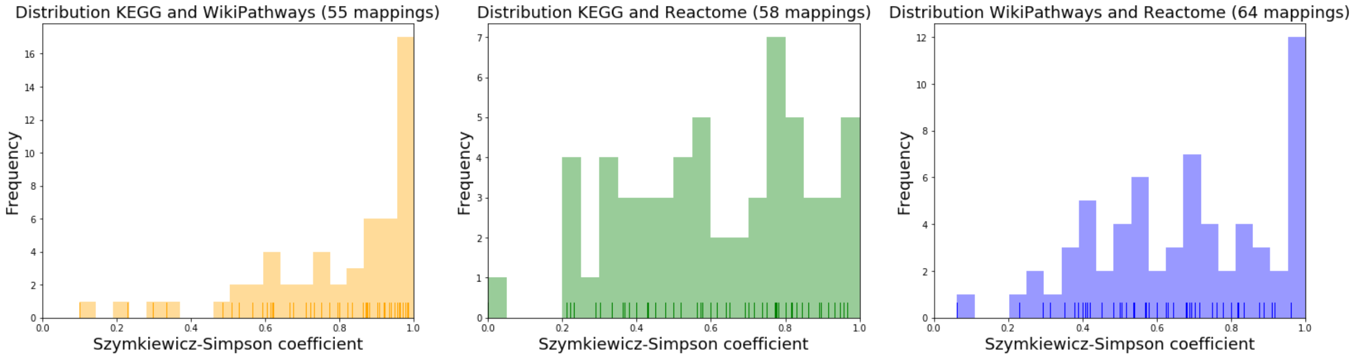

Figure 5. Analysis of the similarity between equivalent pathways in each of the database pairs mapped. The results only present a few outliers with low similarity while most of the mappings comprise highly similar pathways.

The Jupyter notebook that outlines this analysis is located at https://github.com/ComPath/resources/blob/master/notebooks/Mappings%20Similarity%20Analysis.ipynb.

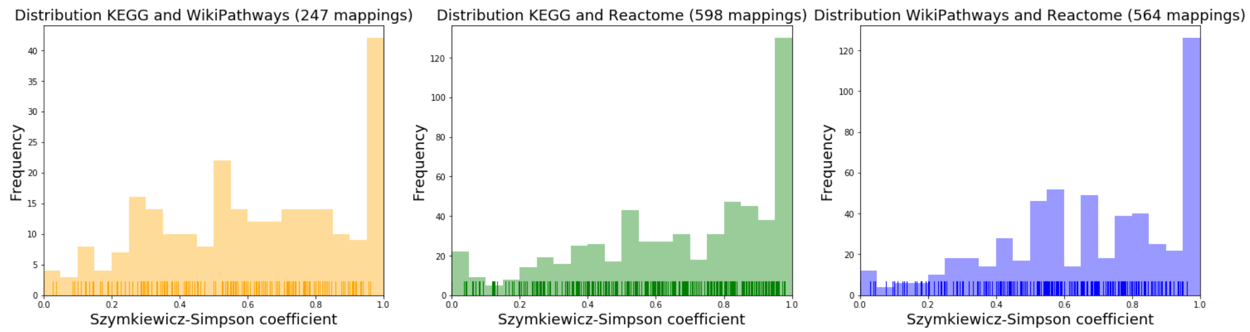

Figure 6. Analysis of the similarity between hierarchical pathways in each of the database pairs mapped. The distributions show how most of the hierarchical mappings share high similarity based on content (left-skewed distribution). The Jupyter notebook that outlines this analysis is located at https://github.com/ComPath/resources/blob/master/notebooks/Mappings%20Similarity%20Analysis.ipynb.

Tables

Resources Loaded in the Deployed ComPath Web Application

**Resource**	**Pathways**	**Genes**	**Version Date**
WikiPathways	438	6015	2018-08-27
Reactome	2195	10633	2018-08-27
KEGG	330	7425	2018-08-27
NeuroMMSig AD	117	903	2018-08-27
NeuroMMSig PD	64	409	2018-08-27

Table 1. Summary of the resources loaded in the deployed ComPath web application. This table is also accessible at https://compath.scai.fraunhofer.de/overview.

Statistics Summary of Pathway Sizes across KEGG, Reactome, and WikiPathways

**Descriptor**	**KEGG**	**Reactome**	**WikiPathways**
Mean	89.63	50.48	42.66
Unbiased variance	8915.80	20841.04	2231.94
Maximum	1273	2729	345
Minimum	1	1	1

Table 2. Statistics of the Pathway Sizes across KEGG, Reactome, and WikiPathways. The Jupyter notebook used to generate this table is available at https://github.com/ComPath/resources/blob/master/notebooks/Statistics%20Pathway%20Sizes.ipynb

Equivalent Pathways within the same Database

Database	Pathway I	Identifier I	Pathway II	Identifier II
WikiPathways	Wnt signaling pathway	WP428	Wnt signaling pathway	WP363
WikiPathways	Notch Signaling Pathway	WP61	Canonical and Non-canonical Notch signaling	WP3845
WikiPathways	Toll-like Receptor Signaling Pathway	WP75	Toll-like Receptor Signaling	WP3858
WikiPathways	Aryl Hydrocarbon Receptor Pathway	WP2873	Aryl Hydrocarbon Receptor	WP2586

Table 3. Equivalent pathways in the same database.

Equivalent Pathways across KEGG, Reactome, and WikiPathways

The Jupyter notebook that outlines this analysis is located at https://github.com/ComPath/resources/blob/master/notebooks/Common%20pathways%20across%20databases.ipynb

KEGG	Reactome	WikiPathways
Mismatch repair	Mismatch Repair	Mismatch repair
Thyroid hormone synthesis	Thyroxine biosynthesis	Thyroxine (Thyroid Hormone) Production
Wnt signaling pathway	Signaling by WNT	Wnt Signaling Pathway
MAPK signaling pathway	MAPK family signaling cascades	MAPK Signaling Pathway
Pentose phosphate pathway	Pentose phosphate pathway (hexose monophosphate shunt)	Pentose Phosphate Pathway
PI3K-Akt signaling pathway	PI3K/AKT activation	PI3K-Akt Signaling Pathway
Toll-like receptor signaling pathway	Toll-Like Receptors Cascades	Toll-like Receptor Signaling Pathway
B cell receptor signaling pathway	B Cell Receptor Signaling Pathway	Signaling by the B Cell Receptor (BCR)
Notch signaling pathway	Signaling by NOTCH	Notch Signaling Pathway
Apoptosis	Apoptosis	Apoptosis
Sphingolipid metabolism	Sphingolipid metabolism	Sphingolipid Metabolism
Hedgehog signaling pathway	Signaling by Hedgehog	Hedgehog Signaling Pathway
Citrate cycle (TCA cycle)	Citric acid cycle (TCA cycle)	TCA Cycle
DNA replication	DNA Replication	DNA Replication
Non-homologous end-joining	Nonhomologous End-Joining (NHEJ)	Non-homologous end joining
Cell cycle	Cell Cycle	Cell Cycle

TGF-beta signaling pathway	Signaling by TGF-beta family members	TGF-beta Signaling Pathway
mTOR signaling pathway	mTOR signalling	Target Of Rapamycin (TOR) Signaling
IL-17 signaling pathway	Interleukin-17 signaling	IL17 signaling pathway
Synthesis and degradation of ketone bodies	Ketone body metabolism	Synthesis and Degradation of Ketone Bodies
Prolactin signaling pathway	Prolactin receptor signaling	Prolactin receptor signaling

Table 4. Equivalent pathways across KEGG, Reactome, and WikiPathways.

Curation examples

Example	Pathway 1	Pathway 2	Mapping
**Same pathway with different context**	AGE-RAGE signaling pathway in diabetic complications (KEGG)	AGE/RAGE pathway (WikiPathways)	No Mapping
**Description provides additional information**	Circadian rhythm related genes (WikiPathways) -	Circadian rhythm (KEGG)	Circadian rythm related genes (WikiPathways) isPartOf Circadian rhythm (KEGG)
**Valid Hierarchical relationship**	Alanine, aspartate and glutamate metabolism (KEGG)	Amino Acid metabolism (WikiPathways)	Alanine, aspartate and glutamate metabolism isPartOf Amino Acid metabolism
**Valid Equivalent mapping**	Mismatch repair (KEGG)	Mismatch Repair (Reactome)	Mismatch repair (KEGG) equivalentTo Mismatch Repair (Reactome)

Table 5. Curation exercise real examples.

Supplementary Text

Case Study II: Additional Findings

M_4 is composed of mitophagy-related pathways and *amyloid fiber formation*, one of the main hypothesis in Alzheimer's disease (AD), in concordance with M_5 that comprises the two pairs of *AD and PD disease pathways* from KEGG and WikiPathways. In contrast to what one could expect, the most similar pathways in this module are the AD and PD KEGG pathways, and not the more natural and expected higher similarity between the two pairs of AD and PD equivalent pathways. Furthermore, the analysis also spotlighted that the WikiPathways *MAPK signaling pathway* from WikiPathways is central between M_2, M_3, M_4; thus, indicating that the three modules might shared cross-talks with MAPK signaling. Finally, the M_6 comprised pathways related to vitamin and cofactors metabolic pathways that also implicated in PD (Etminan *et al.*, 2005; Fariss *et al.*, 2003; De Lau *et al.*, 2006).

Software Installation

All packages described in the manuscript are available through GitHub (https://github.com/) or PyPI (https://pypi.org), the main packaging system for Python 3, under the MIT license. All relevant information for installation is bundled in the package, so it can be easily and quickly installed independently of the operating system, running any modern version of the Python programming language. The documentation for all packages was built using the Python documenting tool Sphinx and is accessible at Read The Docs (https://readthedocs.org).

References

Etminan, M., Gill, S. S., and Samii, A. Intake of vitamin E, vitamin C, and carotenoids and the risk of Parkinson's disease: a meta-analysis. *The Lancet Neurology*, *4*(6), 362-365 (2005).

Fariss, M. W., and Zhang, J. G. Vitamin E therapy in Parkinson's disease. *Toxicology*, *189*(1-2), 129-146 (2003).

De Lau, L. M. L., Koudstaal, P. J., Witteman, J. C. M., Hofman, A., and Breteler, M. M. B. Dietary folate, vitamin B12, and vitamin B6 and the risk of Parkinson disease. *Neurology*, *67*(2), 315-318 (2006).
